# ﻿Additions to the genus *Rhyacopsyche* Müller, 1879 (Trichoptera, Hydroptilidae) in Ecuador

**DOI:** 10.3897/zookeys.1263.148084

**Published:** 2025-12-10

**Authors:** Robin E. Thomson, Blanca Ríos-Touma, Ralph W. Holzenthal

**Affiliations:** 1 Department of Entomology, University of Minnesota, 1980 Folwell Ave., St. Paul, MN, 55108, USA University of Minnesota St. Paul United States of America; 2 Grupo de Investigación en Biodiversidad, Medio Ambiente Ambiente y Salud (BIOMAS), Facultad de Ingenierías y Ciencias Aplicadas, Universidad de Las Américas, Quito CP 170503, Ecuador Universidad de Las Américas Quito Ecuador

**Keywords:** Caddisflies, microcaddisflies, Neotropical, new species, Ochrotrichiinae

## Abstract

Prior to this publication, six species of microcaddisflies in the genus *Rhyacopsyche* (Trichoptera: Hydroptilidae) had been recorded from Ecuador. Herein, we describe five new species: *Rhyacopsyche abdita***sp. nov.**, *Rhyacopsyche bellavista***sp. nov.**, *Rhyacopsyche decouxi***sp. nov.**, *Rhyacopsyche patula***sp. nov.**, and *Rhyacopsyche tumida***sp. nov.** Illustrations of male genitalia are provided with each description. This work brings the total number of *Rhyacopsyche* species in Ecuador to eleven, and the world total to 37.

## ﻿Introduction

The genus *Rhyacopsyche* Müller, 1870 is in the microcaddisfly family Hydroptilidae and subfamily Ochrotrichiinae. The genus is endemic to the New World, occurring primarily in Central and South America, and currently it contains 32 described species (Rocha et al. 2023; Thomson 2023). *Rhyacopsyche peruviana* Flint, 1975 was the first species of this genus recorded from Ecuador (Flint 1975). This single species represented the entirety of our knowledge of the genus in Ecuador until Wasmund and Holzenthal (2007) described *R. benwa*, *R. colubrinosa*, and *R. tanylobosa*. Following that, *R. bunkotala* and *R. hajtoka* were described and recorded from Ecuador by Oláh and Johanson (2011), bringing the total species of Ecuador to six (Ríos-Touma et al. 2017). Besides these last discoveries, aspects of life history are unknown. For the entire genus, *R. mexicana* is the only species with a larval description (Flint 1971). Moreover, there is only one larval record in the global EPTO database (Grigoropoulou et al. 2023), and the record is from Colombia. Explorations of the Trichoptera fauna of Ecuador by Ríos-Touma and colleagues have significantly increased our knowledge of Ecuadorian caddisflies. Aiming to advance knowledge of this genus, we describe five new species of *Rhyacopsyche* in this paper based on material collected in Ecuador, which brings the total number of species known from this genus in the country to 11 (Table 1).

**Table 1.** *Rhyacopsyche* species and distributions within Ecuador (Trichoptera, Hydroptilidae).

Species	Province
*R. abdita* Thomson, Ríos-Touma & Holzenthal, sp. nov.	Imbabura, Morona Santiago, Pichincha
*R. bellavista* Thomson, Ríos-Touma & Holzenthal, sp. nov.	Pichincha
*R. benwa* Wasmund & Holzenthal, 2007	Napo
*R. bunkotala* Oláh & Johanson, 2011	Napo
*R. colubrinosa* Wasmund & Holzenthal, 2007	Cotopaxi, Pastaza, Pichincha, Zamora Chinchipe
*R. decouxi* Thomson, Ríos-Touma & Holzenthal, sp. nov.	Azuay, Bolivar, Imbabura, Pichincha
*R. hajtoka* Oláh & Johanson, 2011	Pichincha
*R. patula* Thomson, Ríos-Touma & Holzenthal, sp. nov.	Pastaza
*R. peruviana* Flint, 1975	Pastaza, Zamora Chinchipe
*R. tanylobosa* Wasmund & Holzenthal, 2007	Morona Santiago, Napo, Pastaza, Pichincha, Santo Domingo, Zamora Chinchipe
*R. tumida* Thomson, Ríos-Touma & Holzenthal, sp. nov.	Azuay

## ﻿Materials and methods

Specimens were prepared and cleared with the use of lactic acid, following the procedure explained in detail by Blahnik et al. (2007). For examination and illustration, cleared genitalia were placed in a watch glass with glycerin and cotton. Cleared genitalia were then observed with an Olympus BX43 compound microscope at 250–500× magnification.

Illustrations and descriptions of prepared specimens followed methods outlined previously in Thomson and Holzenthal (2015). Structures were traced in pencil with the use of an Olympus drawing tube (model U-DA) mounted on the microscope. Pencil sketches were scanned (Fujitsu ScanSnap S1500M scanner) and then edited and digitally inked in Adobe Photoshop and Illustrator (Adobe Creative Cloud v. 6.4.0.361). Each digital “drawing” was completed with the aid of a graphics tablet (Bamboo Pen, Wacom Co., Ltd). Species descriptions were constructed using the program DELTA (Dallwitz et al. 2016).

**Figure 1.**
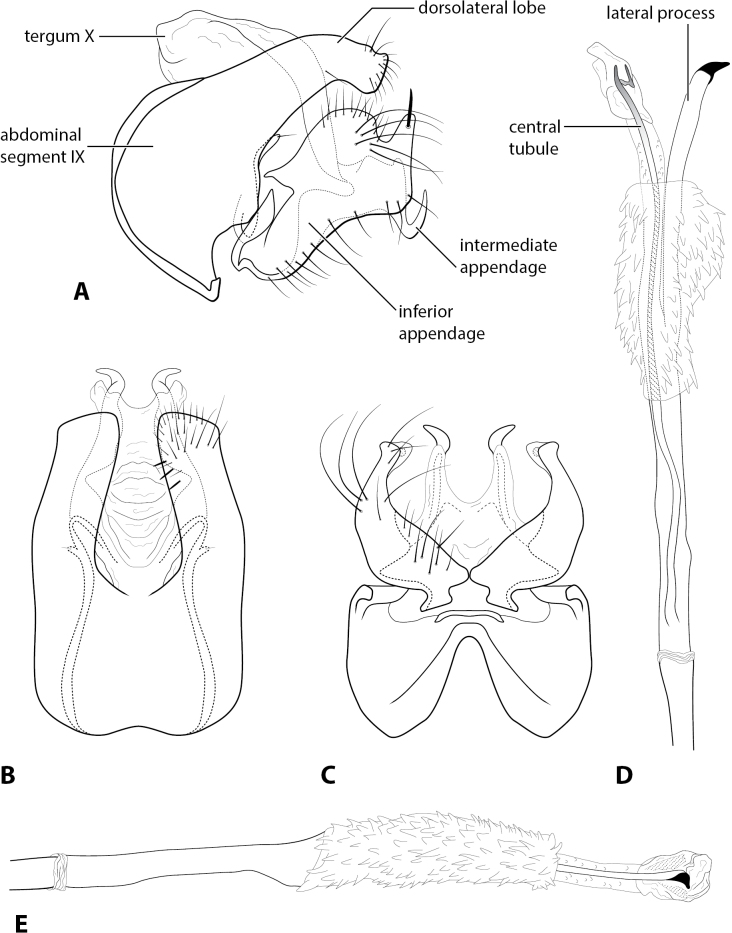
*Rhyacopsyche abdita* sp. nov. (UMSP000113995), male genitalia **A** segments IX–X, lateral **B** segment IX–X, dorsal **C** segment IX, ventral **D** phallus apex, dorsal **E** phallus apex, lateral.

Morphological terminology follows that of Flint (1971) and Marshall (1979), as mirrored by Wasmund and Holzenthal (2007). For simplicity, paired structures are discussed in the singular. Terminology for specific structures is indicated in Fig. 1.

Types and material examined for this study are deposited at the University of Minnesota Insect Collection, Saint Paul, Minnesota, USA (**UMSP**) and the Museo Ecuatoriano de Ciencias Naturales, Quito, Ecuador (**MECN**). Specimen management followed the procedures outlined by Holzenthal and Andersen (2004). Each specimen examined during the study was affixed with a barcode label bearing a unique alphanumeric sequence beginning with the prefix UMSP. Specimen-level taxonomic, locality, collection, and other information are stored in the University of Minnesota Insect Collection database using the software Specify 7 (Specify Collections Consortium 2023) and made available through the Global Biodiversity Information Facility (GBIF 2025).

## ﻿Species descriptions

### ﻿ Rhyacopsyche abdita sp. nov.

Taxon classificationAnimaliaTrichopteraHydroptilidae

268CA781-939D-5239-95CB-B01ADC8A9234

https://zoobank.org/2BE02EC5-3BE5-4364-969F-122B9484D5F8

Fig. 1

#### Type material.

***Holotype***: Ecuador • ♂; Morona-Santiago, trib. to Rio Abanico, Hwy E46 (via Riobamba-Macas); 02.24985°S, 078.20238°W; el. 1531 m; 26 January 2015; Holzenthal, Huisman, Ríos-Touma, Amigo leg.; in alcohol; UMSP000113995; UMSP. ***Paratypes***: Ecuador • 1 ♂; Pichincha, Reserva Rio Bravo, Rio Bravo, upstream; 0.07802°S, 078.73287°W; 19 November 2023; el. 1564 m; Holzenthal, Frandsen, Morabowen, Amigo leg.; pan trap; in alcohol; UMSP000551404; UMSP • 5 ♂; Imbabura, Reserva Los Cedros, tributary to Rio Los Cedros; 00.30374°N, 078.78229°W; el. 1312 m; 18–19 October 2011 Holzenthal, Ríos, Encalada, Acosta leg.; in alcohol; UMSP000145862; MECN • 7 ♂; same as for preceding; UMSP000145861; UMSP • 8 ♂; Imbabura, Reserva Los Cedros, Rio de la Plata; 00.32495°N, 078.78084°W; el. 1587 m; 18 October 2011; Holzenthal, Ríos, Encalada, Acosta leg.; in alcohol; UMSP000520782; UMSP • 1 ♂; Imbabura, Reserva Los Cedros, small stream near station; 00.31127°N 78.78150°W; el. 1460 m; 17 October 2011; Holzenthal, Ríos, Encalada, Acosta leg.; in alcohol; UMSP000138550; MECN.

#### Diagnosis.

The form and appearance of the dorsolateral lobe, inferior appendage, and phallus of *Rhyacopsyche abdita*, new species, and *R. intraspira* Wasmund & Holzenthal, 2007 are similar. However, the prominent hook at the apex of the intermediate appendage of *R. abdita*, which is lacking in *R. intraspira*, can be used to distinguish between the two. Additionally, *R. abdita* has far fewer of the dark, peg-like setae on the dorsolateral lobe than *R. intraspira*.

#### Description.

***Adult*** (male: *n* = 23). Forewing length 3.5–3.8 mm (male). Head unmodified, with 3 ocelli; antennae unmodified. Tibial spurs 1, 3, 4. Specimens brown in alcohol. Sternum VII with short mesoventral point. ***Male genitalia*.** Abdominal segment IX setose, anterolateral margin convex, posterolateral margin with mesal hump and bearing small digitate process with apical seta on inner surface; dorsolateral lobe of segment IX truncate and setose, in dorsal view deeply divided and with small peg-like setae on inner surface (Fig. 1A, B). Tergum X membranous, partially retracted inside segment IX, articulating with intermediate appendage (Fig. 1A). Intermediate appendage elongate, extending past inferior appendage, apex hook-like (Fig. 1A, B). Inferior appendage setose, dorsal margin enlarged mesally, ventral margin sinuate; apex bearing prominent, peg-like seta (Fig. 1A). Phallus basally tubular, elongate, thin membranous sheath surrounding middle portion covered with spicules; central tubule with preapical, geniculate projection; lateral process with darkened, hooked apex (Fig. 1D, E).

#### Etymology.

*Abditus*, Latin for “hidden, concealed,” referring to the digitate projections on the inner surface of abdominal segment IX.

#### Distribution.

Ecuador (Table 1).

### ﻿ Rhyacopsyche bellavista sp. nov.

Taxon classificationAnimaliaTrichopteraHydroptilidae

7B28FD02-2049-5C37-839F-624F619A37A0

https://zoobank.org/CEDF9ADF-5745-465E-A420-82F96FAD6F5A

Fig. 2

#### Type material.

***Holotype***: Ecuador • ♂; Pichincha, Bellavista Cloud Forest Reserve and Lodge, small stream; 00.01212°S, 078.68958°W; 13 July 2017; el. 2614 m; Andrea Tapia leg.; pan trap; in alcohol; UMSP000560013; UMSP. ***Paratypes***: Ecuador • 1 ♂ and 1 ♀; same collection data as for holotype; 15 July 2017; in alcohol; UMSP000560011; MECN.

#### Diagnosis.

*Rhyacopsyche bellavista*, new species, is most similar to *R. rhamphisa* Wasmund & Holzenthal, 2007; both species have a slender inferior appendage that curves dorsad and terminates in a strongly sclerotized apex that points mesally. The inferior appendage curves much more strongly in *R. bellavista* than it does in *R. rhamphisa*. The general appearance of tergum X in *R. bellavista*, large and bearing apical spines and spicules apically, can also be used to diagnose *R. bellavista*.

#### Description.

**Figure 2.**
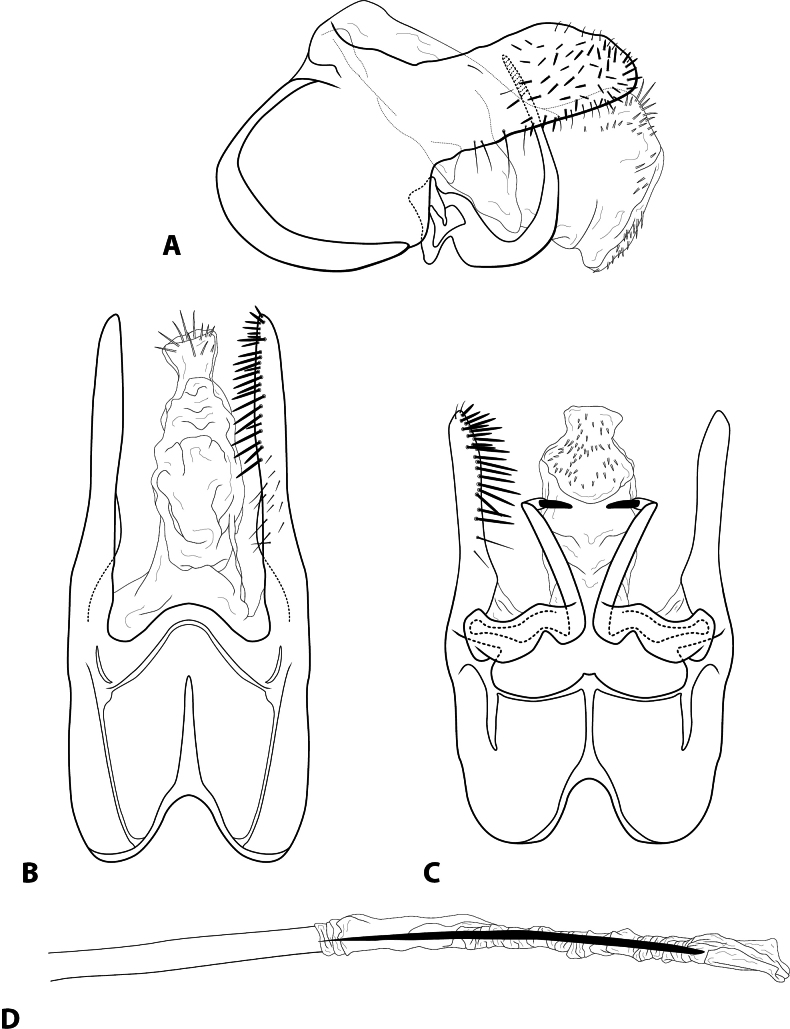
*Rhyacopsyche bellavista* sp. nov. (UMSP000560013), male genitalia **A** segments IX–X, lateral **B** segment IX–X, dorsal **C** segments IX–X, ventral **D** phallus apex, dorsal.

***Adult*** (male: *n* = 2, female: *n* = 1). Forewing length 3.8–3.9 mm (male); 3.9 mm (female). Head unmodified, with 3 ocelli; antennae unmodified. Tibial spurs 1, 3, 4. Specimens brown in alcohol. Sternum VII with short mesoventral point. ***Male genitalia*.** Abdominal segment IX setose, anterolateral margin rounded, posterolateral margin short, straight; dorsolateral lobe of segment IX large, straight, directed slightly dorsad, bearing dark, peg-like setae, in dorsal view deeply and broadly divided, slender (Fig. 2A, B). Tergum X membranous, partially retracted inside segment IX; apex large, extending past both dorsolateral lobe of segment IX and inferior appendage, with spines and spicules apically; in dorsal view, slender (Fig. 2A, B). Intermediate appendage not apparent. Inferior appendage broadest basally, simple, elongate, slender, curving strongly dorsad; apex strongly sclerotized, pointed, bent mesad (Fig. 2A, C). Phallus basally tubular, elongate, apex membranous; central tubule pointed; lateral process not apparent (Fig. 2D).

#### Etymology.

Named for the reserve where the type specimens were collected.

#### Distribution.

Ecuador (Table 1).

### ﻿ Rhyacopsyche decouxi sp. nov.

Taxon classificationAnimaliaTrichopteraHydroptilidae

391D4339-50BD-580D-820E-72E858B567ED

https://zoobank.org/D06CFD09-9C32-441B-91AA-2189DF10DA37

Fig. 3

#### Type material.

***Holotype***: Ecuador • ♂; Imbabura, Reserva Los Cedros, tributary to Rio Los Cedros; 00.30374°N, 078.78229°W; el. 1312 m; 18–19 October 2011; Holzenthal, Ríos, Encalada, Acosta leg.; in alcohol; UMSP000145864; UMSP. ***Paratypes***: Ecuador • 4 ♂; same as for holotype; in alcohol; UMSP000145864; MECN • 7 ♂ and 2 ♀; same as for holotype; in alcohol; UMSP000145868, UMSP00145869; UMSP • 1 ♂; Azuay, Rio Chaucha & waterfall 1.2 km E La Iberia; 02.89407°S, 079.50786°W; el. 700 m; 22 March 2022; Ríos, Pauls, Amigo leg.; in alcohol; UMSP000418196; MECN • 1 ♂; Azuay, trib. to Rio Chaucha in San Antonio de Chaucha; 02.90736°S, 079.41375°W; el. 1831 m; 23 March 2022; Holzenthal, Ríos, Amigo leg.; in alcohol; UMSP000418195; UMSP • 1 ♂; Bolivar, Quebrada San Pablo, N of Chazojuan; 01.37978°S, 079.15496°W; el. 1150 m; 20 March 2022; Ríos, Holzenthal, Pauls, Thomson, Amigo leg.; in alcohol; UMSP000418194; UMSP • 1 ♂; Pichincha, Reserva Rio Bravo, Rio Bravo, upstream; 00.07802°S, 078.73287°W; 19 November 2023; el. 1564 m; Holzenthal, Frandsen, Morabowen, Amigo leg.; pan trap; in alcohol; UMSP000551405; UMSP.

#### Diagnosis.

*Rhyacopsyche decouxi*, new species, is also similar in appearance to *R. rhamphisa*. Characteristics found on the dorsolateral lobe, inferior appendage, and phallus make them very similar. Key differences, however, make it possible to separate the two. *R. decouxi* bears a prominent seta on the dorsal margin of the inferior appendage that is lacking in *R. rhamphisa*; in addition, the shape of the inferior appendage when viewed ventrally is very different.

#### Description.

**Figure 3.**
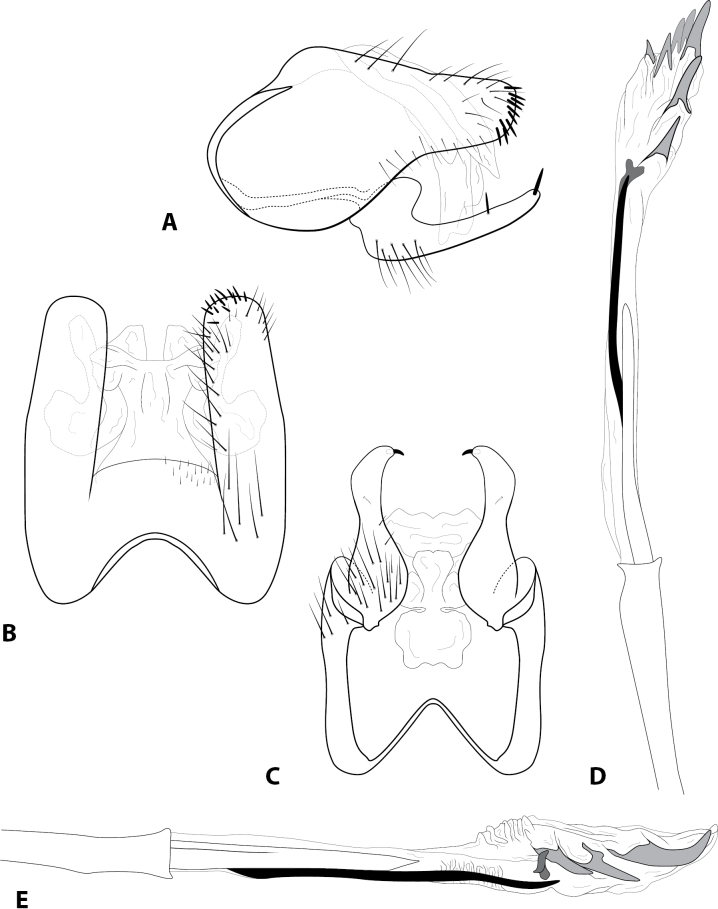
*Rhyacopsyche decouxi* sp. nov. (UMSP000145864), male genitalia **A** segments IX–X, lateral **B** segment IX–X, dorsal **C** segments IX–X, ventral **D** phallus apex, dorsal **E** phallus apex, lateral.

***Adult*** (male: *n* = 15, female: *n* = 2). Forewing length 2.5–2.9 mm (male), 2.9–3.0 mm (female). Head unmodified, with 3 ocelli; antennae unmodified. Tibial spurs 1, 3, 4. Specimens brown in alcohol. Sternum VII with short mesoventral point. ***Male genitalia*.** Abdominal segment IX setose, anterolateral margin rounded, posterolateral margin convex; dorsolateral lobe of segment IX broadly truncate, with dark, peg-like setae, in dorsal view deeply divided and with rounded apex (Fig. 3A, B). Tergum X membranous, retracted inside segment IX, in dorsal view with lateral lobes (Fig. 3A, B). Intermediate appendage not apparent. Inferior appendage elongate, setose basally, dorsal margin produced basally and with prominent seta mesally; apex digitate, bearing prominent peg-like seta (Fig. 3A, C). Phallus basally tubular, elongate, membranous apex bearing series of thickened spines; central tubule with pointed apex adjacent to small, sclerotized plate; lateral process lanceolate (Fig. 3D, E).

#### Etymology.

Named in honor of the late José Decoux, founder and protector of the reserve, who died in 2024, leaving the enormous legacy of Los Cedros Reserve to future generations.

#### Distribution.

Ecuador (Table 1).

### ﻿ Rhyacopsyche patula sp. nov.

Taxon classificationAnimaliaTrichopteraHydroptilidae

85314003-3193-5BE6-886F-79C48A44C8BD

https://zoobank.org/A5BC6702-36E8-4C33-AE83-4154B80A9CF5

Fig. 4

#### Type material.

***Holotype***: Ecuador • ♂; Pastaza, small stream, ca 3.8 km (rd) SE Chuwitayo; 01.92251°S, 077.79459°W; el. 703 m; 20 September 2021; Ríos, Holzenthal, Frandsen, Errigo, Amigo leg.; in alcohol; UMSP000280912; MECN.

#### Diagnosis.

*Rhyacopsyche patula*, new species, is most similar to *R. holzenthali* Harris & Armitage, 2019. Both species are lacking the deep division often observed in the posterior margin of the dorsolateral lobe when viewed dorsally, and the general appearance of the inferior appendage is very similar between the two when viewed laterally. However, *R. patula* is lacking both the small emargination and the peg-like setae present on the dorsolateral lobe of *R. holzenthali*. In addition, the inferior appendage of *R. holzenthali* is more slender than that of *R. patula*.

#### Description.

**Figure 4.**
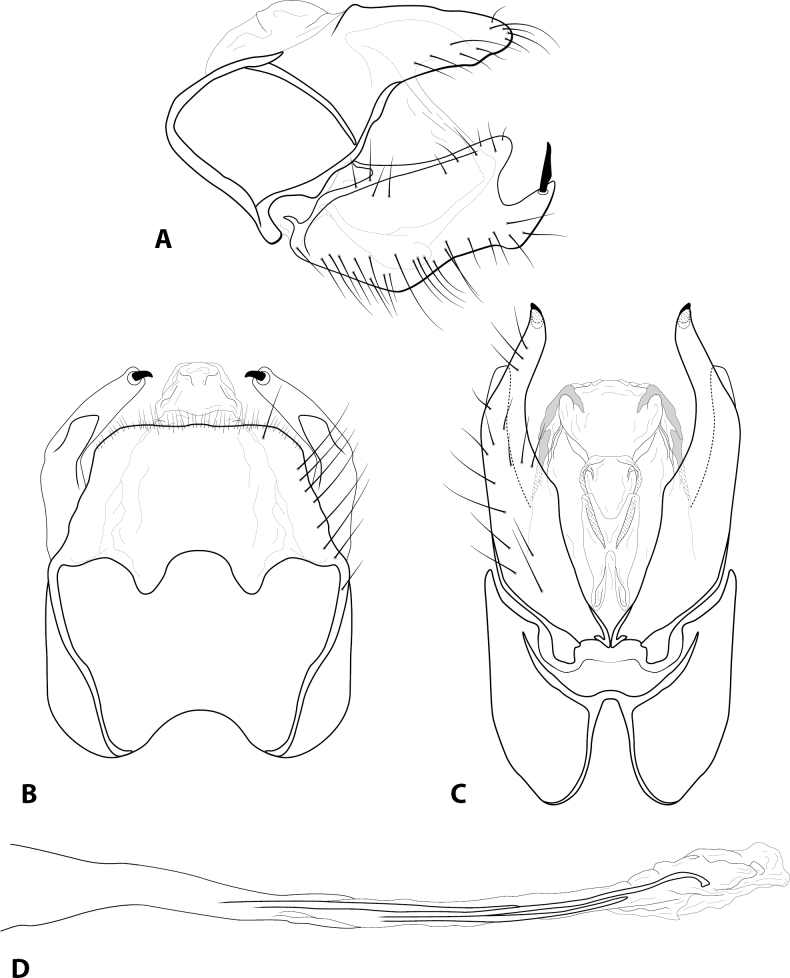
*Rhyacopsyche patula* sp. nov. (UMSP000280912), male genitalia **A** segments IX–X, lateral **B** segment IX–X, dorsal **C** segment IX, ventral **D** phallus, dorsal.

***Adult*** (male: *n* = 1). Forewing length 2.9 mm (male). Head unmodified, with 3 ocelli; antennae unmodified. Tibial spurs 1, 3, 4. Specimen brown in alcohol. Sternum VII with short mesoventral point. ***Male genitalia*.** Abdominal segment IX setose, anterolateral margin rounded, posterolateral margin irregular and with ventral projection; dorsolateral lobe of segment IX setose, rounded, in dorsal view broad and truncate (Fig. 4A, B). Tergum X membranous, partially retracted inside segment IX, fused with intermediate appendage (Fig. 4A, B). Intermediate appendage membranous, fused mesally, with lightly sclerotized sections laterally and ventrally (Fig. 4A, C). Inferior appendage setose, dorsal margin enlarged mesally, ventral margin sinuate; apex bearing prominent, peg-like seta (Fig. 4A, C). Phallus basally tubular, elongate, apex membranous; central tubule with slight hooked apex in dorsal view; lateral process pointed (Fig. 4D).

#### Etymology.

*Patulus*, Latin for “spread out, broad,” referring to the broad dorsolateral lobe of abdominal segment IX, without division when viewed dorsally.

#### Distribution.

Ecuador (Table 1).

### ﻿ Rhyacopsyche tumida sp. nov.

Taxon classificationAnimaliaTrichopteraHydroptilidae

9C7C3CF7-E91C-56EA-8DE5-C488B0A0697C

https://zoobank.org/8CA6CDDF-F8B0-400B-9D00-D380B4387AAC

Fig. 5

**Type material. *Holotype***: Ecuador • ♂; Azuay, Rio Balao Grande in El Progreso [Sta. Isabel]; 02.90989°S, 079.59665°W; el. 213 m; 21 March 2022; Ríos, Holzenthal, Pauls, Frandsen, Thomson, Amigo leg.; in alcohol; UMSP000280911; UMSP. ***Paratypes***: Ecuador • 1 ♂; Azuay, Rio Chaucha & waterfall, 1.2 km E La Iberia; 02.89407°S, 079.50786°W; el. 700 m; 22 March 2022; Ríos, Pauls, Amigo leg.; in alcohol; UMSP000418191; UMSP • 1 ♂; same as for preceding; UMSP000418192; MECN.

#### Diagnosis.

This species most closely resembles *R. turrialbe* Flint, 1971, based on the overall form and appearance of abdominal segment IX and the inferior appendage. It can be most easily distinguished by the inferior appendage, which curves outward at the apex in *R. tumida* rather than curling inwards as in *R. turrialbe*.

#### Description.

**Figure 5.**
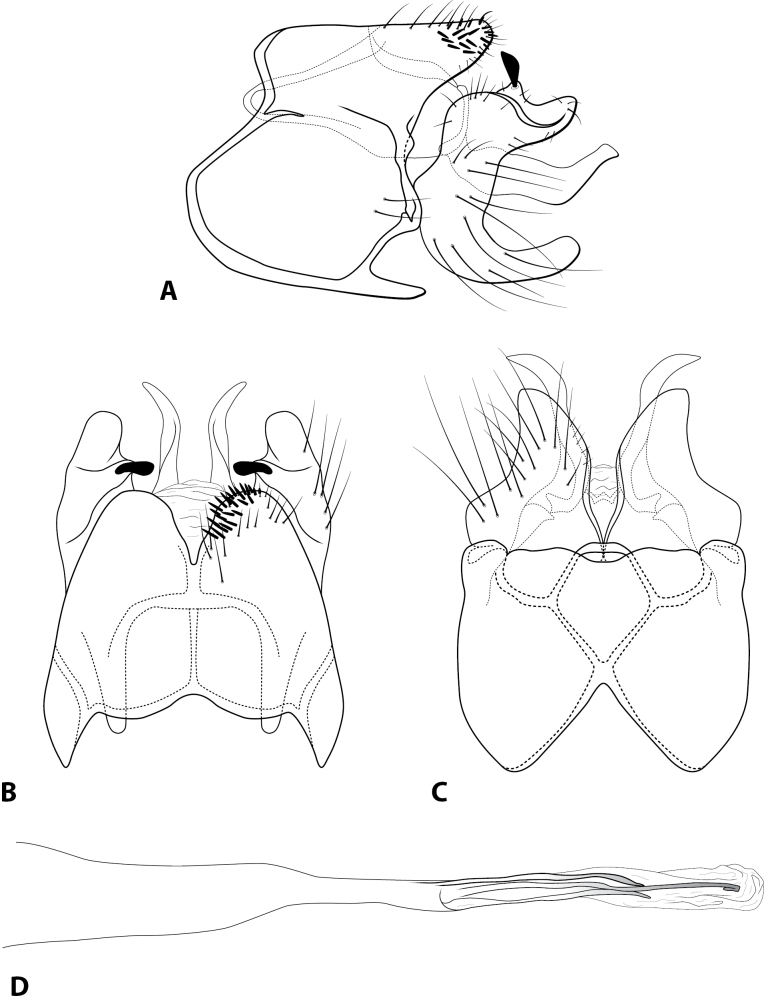
*Rhyacopsyche tumida* sp. nov. (UMSP000280911), male genitalia **A** segments IX–X, lateral **B** segment IX–X, dorsal **C** segment IX, ventral **D** phallus, dorsal.

***Adult*** (male: *n* = 3). Forewing length 3.3–3.5 mm (male). Head unmodified, with 3 ocelli; antennae unmodified. Tibial spurs 1, 3, 4. Specimens brown in alcohol. Sternum VII with short mesoventral point. ***Male genitalia*.** Abdominal segment IX setose, anterolateral margin sinuate, posterolateral margin irregular with ventral projection; dorsolateral lobe of segment IX rounded with dark, peg-like setae, in dorsal view divided and rounded (Fig. 5A, B). Tergum X membranous, retracted inside segment IX, with basal projections, articulating with intermediate appendage (Fig. 5A, B). Intermediate appendage elongate, extending posteriad and bent dorsad, in lateral view apex curving slightly outward (Fig. 5A, B). Inferior appendage wide, setose, divided into dorsal and ventral lobes; dorsal lobe with posterior margin produced into blunt process, dorsal margin bearing large, bulbous, peg-like seta; ventral lobe simple, broadly rounded (Fig. 5A). Phallus basally tubular, elongate, apex membranous; central tubule with hooked apex; lateral process pointed (Fig. 5D).

#### Etymology.

*Tumidus*, Latin for “swollen,” referring to the shape of the thick seta on the dorsal lobe of the inferior appendage.

#### Distribution.

Ecuador (Table 1).

## ﻿Discussion

This work nearly doubles the number of species within the genus *Rhyacopsyche* (Trichoptera, Hydroptilidae) known from Ecuador from six to 11. All five of the new species described here are currently known only from collections made in Ecuador.

When compared to neighboring countries, Peru also has 11 recorded *Rhyacopsyche* species, while Colombia has only six (Table 2) (Ríos-Touma et al. 2017; Rocha et al. 2023; Thomson 2023). Interestingly, there is no overlap in the recorded species found between Colombia and Ecuador, while Ecuador and Peru share five species in common. This likely reflects the differing levels of surveying intensity that has occurred in each country and indicates that more species are likely to be found in all three.

**Table 2.** *Rhyacopsyche* species recorded from Ecuador and neighboring countries Colombia and Peru.

Species	Country		
	Colombia	Ecuador	Peru
*R. abdita* Thomson, Ríos-Touma & Holzenthal, sp. nov.		X	
*R. andina* Flint, 1991	X		X
*R. bellavista* Thomson, Ríos-Touma & Holzenthal, sp. nov.		X	
*R. benwa* Wasmund & Holzenthal, 2007		X	X
*R. bunkotala* Oláh & Johanson, 2011		X	
*R. colombiana* Wasmund & Holzenthal, 2007	X		
*R. colubrinosa* Wasmund & Holzenthal, 2007		X	X
*R. decouxi* Thomson, Ríos-Touma & Holzenthal, sp. nov.		X	
*R. escamosa* Rocha, Santos & Nessimian, 2023			X
*R. hajtoka* Oláh & Johanson, 2011		X	X
*R. hasta* Wasmund & Holzenthal, 2007			X
*R. intraspira* Wasmund & Holzenthal, 2007			X
*R. jimena* Flint, 1991	X		
*R. matthiasi* Flint, 1991	X		
*R. mutisi* Mey & Joost, 1990	X		
*R. patula* Thomson, Ríos-Touma & Holzenthal, sp. nov.		X	
*R. peruviana* Flint, 1975		X	X
*R. rhamphisa* Wasmund & Holzenthal, 2007	X		X
*R. tanylobosa* Wasmund & Holzenthal, 2007		X	X
*R. tumida* Thomson, Ríos-Touma & Holzenthal, sp. nov.		X	
*R. yungas* Rocha, Santos & Nessimian, 2023			X

Moreover, only one larva has been collected from this genus in Ecuador (Ríos-Touma pers. comm.). These observations highlight the need for an increase in taxonomic research focused on the insect fauna of not just Ecuador specifically, but South America in general.

## Supplementary Material

XML Treatment for Rhyacopsyche abdita

XML Treatment for Rhyacopsyche bellavista

XML Treatment for Rhyacopsyche decouxi

XML Treatment for Rhyacopsyche patula

XML Treatment for Rhyacopsyche tumida

## ﻿Additional information

### Author ORCIDs

Robin E. Thomson https://orcid.org/0000-0002-5883-2040

Blanca Ríos-Touma https://orcid.org/0000-0002-3921-0908

Ralph W. Holzenthal https://orcid.org/0000-0003-1853-6340

### Data availability

All of the data that support the findings of this study are available in the main text.
